# Death Receptor 3 Signaling Controls the Balance between Regulatory and Effector Lymphocytes in SAMP1/YitFc Mice with Crohn’s Disease-Like Ileitis

**DOI:** 10.3389/fimmu.2018.00362

**Published:** 2018-03-01

**Authors:** Zhaodong Li, Ludovica F. Buttó, Kristine-Anne Buela, Li-Guo Jia, Minh Lam, John D. Ward, Theresa T. Pizarro, Fabio Cominelli

**Affiliations:** ^1^BRB-5, Digestive Health Research Institute, Case Western Reserve University, Cleveland, OH, United States; ^2^Department of Pathology, Case Western Reserve University, Cleveland, OH, United States

**Keywords:** Crohn’s disease, inflammatory bowel disease, death receptor 3, SAMP1/YitFc, ileitis, regulatory T cells, innate lymphoid cell, TL1A, CD25^+/−^ T cells

## Abstract

Death receptor 3 (DR3), a member of the tumor necrosis factor receptor (TNFR) superfamily, has been implicated in regulating T-helper type-1 (T_H_1), type-2 (T_H_2), and type-17 (T_H_17) responses as well as regulatory T cell (T_reg_) and innate lymphoid cell (ILC) functions during immune-mediated diseases. However, the role of DR3 in controlling lymphocyte functions in inflammatory bowel disease (IBD) is not fully understood. Recent studies have shown that activation of DR3 signaling modulates T_reg_ expansion suggesting that stimulation of DR3 represents a potential therapeutic target in human inflammatory diseases, including Crohn’s disease (CD). In this study, we tested a specific DR3 agonistic antibody (4C12) in SAMP1/YitFc (SAMP) mice with CD-like ileitis. Interestingly, treatment with 4C12 prior to disease manifestation markedly worsened the severity of ileitis in SAMP mice despite an increase in FoxP3^+^ lymphocytes in mesenteric lymph node (MLN) and small-intestinal lamina propria (LP) cells. Disease exacerbation was dominated by overproduction of both T_H_1 and T_H_2 cytokines and associated with expansion of dysfunctional CD25^−^FoxP3^+^ and ILC group 1 (ILC1) cells. These effects were accompanied by a reduction in CD25^+^FoxP3^+^ and ILC group 3 (ILC3) cells. By comparison, genetic deletion of DR3 effectively reversed the inflammatory phenotype in SAMP mice by promoting the expansion of CD25^+^FoxP3^+^ over CD25^−^FoxP3^+^ cells and the production of IL-10 protein. Collectively, our data demonstrate that DR3 signaling modulates a multicellular network, encompassing T_regs_, T effectors, and ILCs, governing disease development and progression in SAMP mice with CD-like ileitis. Manipulating DR3 signaling toward the restoration of the balance between protective and inflammatory lymphocytes may represent a novel and targeted therapeutic modality for patients with CD.

## Introduction

Crohn’s disease (CD) is an inflammatory bowel disease (IBD) characterized by chronic and relapsing inflammation of gut intestinal segments. Although the cause of the disease is still unknown, an exaggerated immune response against commensal bacteria in individuals with a genetic predisposition has been postulated as a key mechanism (1). Pharmacological treatment of the disease is generally based upon suppression of the immune system using non-specific drugs and blockade of inflammatory processes by biological therapy, such as antibodies targeting the cytokine TNF-α (2). However, a significant percentage of patients fail to improve or maintain remission for prolonged periods. For these individuals very limited options currently exist. As a result, more than 70% of patients require surgical removal of the affected intestinal segments. Furthermore, surgery does not necessarily provide long-lasting resolution of the inflammatory process, and recurrence after surgery occurs in the majority of patients with CD (3). Thus, to date there remains no cure for this devastating disease.

Death receptor 3 (DR3) (TNFRSF25), a member of TNFR superfamily expressed primarily on lymphocytes and innate lymphoid cells (ILCs), is a receptor for the cytokine TL1A (TNFSF15) secreted by dendritic cells, monocytes, macrophages, plasma cells, synovial fibroblasts, and endothelial cells (4–12). Preclinical and clinical studies have clearly shown a fundamental role for the TL1A/DR3 cytokine/receptor pair in the pathogenesis of inflammatory diseases, including rheumatoid arthritis (13–15), diabetic retinopathy (16), pulmonary sarcoidosis (17), asthma (10, 18), and, especially, IBD (19). Precisely, TL1A and DR3 expression is significantly increased, in an inflammation-specific manner, in both serum and inflamed tissues in IBD patients and in murine experimental ileitis (19). Genome-wide association studies have identified polymorphisms associated with IBD risk in the gene that encodes for TL1A protein (20–24). Finally, studies in animal models of intestinal inflammation have demonstrated that sustained expression of TL1A leads to chronic small-intestinal inflammation, whereas blockade of the TL1A/DR3 axis suppresses murine colitis (7, 12). Our laboratory has previously identified a novel role of TL1A/DR3 system in modulating lymphocyte functions and in preserving gut homeostasis in dextran sodium sulfate (DSS)-induced acute colitis (25). Specifically, following DSS treatment, TL1A- and DR3-deficient mice displayed an increase in disease severity mediated by defective suppressive function of regulatory T cells (T_regs_), and a concomitant expansion of pro-inflammatory T-helper type-1 (T_H_1), type-2 (T_H_2), and type-17 (T_H_17) (25). These results provided compelling evidence that TL1A/DR3 signaling exerts pleiotropic effects on lymphocyte homeostasis, cell proliferation, activation, function, and differentiation, mediating the balance between inflammatory and T_reg_ responses. Additional data supporting the role of DR3 in T_reg_ functionality consist in the observation that treatment with 4C12, an agonistic antibody to DR3, induces selective expansion of T_regs_ and reduces activation of conventional T cells in an allergic lung mouse model (26), in cardiac allografts (27) and in graft vs. host disease mouse model (28). This demonstrates that modulation of DR3 signaling may be a potential therapeutic target in immune-mediated disease, hence leading to appealing applications in CD therapy.

Recent findings have demonstrated that DR3-expressing ILCs could be an integral part of the DR3 signaling network (8, 10, 11). As effectors of innate immunity and regulators of tissue modeling, ILCs have been shown to play an important role in inflammatory diseases in the skin, lung, and gut (29). It is thought that the identified ILC populations, including group 1 (ILC1), group 2 (ILC2), and group 3 (ILC3), have a cytokine expression pattern that resembles that of T_H_1, T_H_2, and T_H_17/T_H_22 cells, respectively (30). ILC1 subset, found enriched in inflamed intestine of CD patients, expresses the transcription factor T-bet and responds to interleukin 12 (IL-12) by producing interferon-γ (IFN-γ) (31–33). The development and function of ILC2 cells depend on the transcription factor Gata-3 and produce the type-2 cytokines IL-5 and IL-13. The important role of ILC2s in virus-induced experimental models of airway hyperactivity and in allergic lung responses has been recognized (34–36). Regulated by the transcription of retinoic acid receptor-related orphan receptor-γt (RORγt), ILC3s produce IL-17 and IL-22 in response to IL-23 and IL-1β (37–39). ILC3s play a protective role in the healthy gut, by modulating epithelial cell regeneration through IL-22 secretion (40, 41). Nevertheless, innate sources of IL-17 were found significantly elevated in the intestinal mucosa of CD and Ulcerative colitis (UC) patients, suggesting a contribution of ILC3s to intestinal inflammation in IBD (38).

Recent data from our group supports a pro-inflammatory role of DR3/TL1A signaling mediated through activation of effector T cells during chronic inflammation, underscoring the importance of this cytokine–receptor pair in promoting gut immunopathology (Cominelli *et al*., unpublished data). However, the discovery that DR3 promotes T_reg_ expansion (26–28) has led to the hypothesis of whether T_reg_ proliferation prior to disease initiation can revert CD-like ileitis. Therefore, in the current work, we investigated whether treatment with 4C12 prior to disease onset could delay or even ablate ileitis in SAMP mice, and whether DR3 is a master regulator of lymphocyte functions. We evaluated the distribution of total T_regs_ (CD4^+^FoxP3^+^), of CD25^+^ and CD25^−^ T_reg_ subsets, and of ILCs in mesenteric lymph node (MLN) and lamina propria (LP) cells. Interestingly, our results indicate that DR3 stimulation accelerates and exacerbates ileitis onset by triggering T_H_1 and T_H_2 responses, and by mitigating anti-inflammatory processes. In addition, our data suggest that DR3 signaling pathway promotes the expansion of non-regulatory CD25^−^ T cells and ILC1s concomitant to the reduction of CD25^+^ T_regs_ and ILC3s.

## Materials and Methods

### Antibodies and Reagents

Agonistic anti-DR3 (4C12) monoclonal Ab and control Armenian Hamster IgG isotype (IgG) were purchased from BioLegend (San Diego, CA, USA). Anti-CD3e (2C11), anti-CD28 (37.51), anti-IL-17A (TC11-18H10), and CD16/CD32 (2.4G2) Abs were purchased from BD Biosciences (San Diego, CA, USA). T_regs_ were stained by using the FoxP3^+^T_reg_ staining kit following the manufacturer’s instructions (eBioscience, San Diego, CA, USA). Collagenase and DNase were obtained from Sigma-Aldrich (St. Louis, MO, USA), and dispase from Roche (Mannheim, Germany). RPMI-1640 cell culture medium (RPMI), fetal bovine serum (FBS), penicillin, and streptomycin (P/S) were all purchased from Invitrogen (Grand Island, NY, USA). Cytokines and other reagents were purchased from the following vendors: TGF-β1 and IL-6 (R&D Systems, Minneapolis, MN, USA), IL-2 (eBioscience), and PMA, ionomycin and GolgiStop (BD Biosciences). All ELISA kits were purchased from eBioscience.

### Experimental Animals

An equal number of male and female 5-week-old SAMP and age/gender-matched AKR/J (AKR) mice, and 10-week-old SAMP × DR3^−/−^ (DR3_KO_) and age/gender-matched SAMP × DR3^+/+^ (DR3_WT_) mice were used in each experiment, with a mean body weight of 26.3 g on the day of sacrifice. Mice were housed and maintained in ventilated micro-isolator cages (Allentown Inc.) with 1/8-inch corn bedding and cotton nestlets for environmental enrichment (Envigo), kept on 12-h light/dark cycles, and maintained under specific-pathogen-free conditions in the Animal Resource Center of Case Western Reserve University (CWRU). All mice had *ad libitum* access to water and were fed with standard laboratory rodent diet P3000 (Harlan Teklad) throughout the experiments. Mice were genotyped by PCR-based assays of genomic tail DNA. All experimental procedures were approved by the Institutional Animal Care and Use Committee of CWRU and were in accordance with the Association for Assessment and Accreditation of Laboratory Animal Care guidelines. All experiments were conducted in a blinded manner, without prior knowledge of treatments and mouse groups by the experimenter. Mice were randomized to different interventions using a progressive numerical number. The code for each mouse was known only to the animal caretaker and was revealed at the end of the study.

### Treatment

Five-week-old SAMP and AKR mice were given intraperitoneal injections of 10 µg of 4C12 (or IgG) in 100-µL PBS, weekly, for 4 weeks, as previously described elsewhere (26). Mice were sacrificed at the beginning of the fifth week.

### Histology

Mouse ilea were collected, rinsed with phosphate-buffered saline (PBS), fixed in Bouin’s fixative solution (Fisher Scientific, Pittsburgh, PA, USA), embedded in paraffin, and sectioned. Histological evaluation of inflammation severity was determined in hematoxylin and eosin-stained 5-μm-thick sections, by using a semi-quantitative scoring system as previously described (42). Briefly, scores ranging from 0 (normal histology) to 3 (maximum severity of histologic changes) were used to evaluate histologic indices for (1) active inflammation (infiltration with neutrophils), (2) chronic inflammation (lymphocytes and plasma cells in the mucosa and submucosa), (3) monocyte inflammation (macrophages in the mucosa and submucosa), (4) villous distortion (flattening and/or widening of normal villus architecture), and (5) transmural inflammation. The total inflammatory index represents the sum of all five individual components. Histological scoring was performed by a single trained pathologist in a blinded fashion.

### Stereomicroscopy

Ileal tissue abnormalities (i.e., cobblestone lesions) and normal mucosa were investigated by examining the cellular structural pattern of ileal tissue *via* stereomicroscopy, cm by cm, using a reference catalogue of lesions, as previously described (43). Starting from the distal end, 10 cm of ileum were collected, fixed in Bouin’s solution overnight, and then transferred to 70% ethanol for stereomicroscopic analysis. Both healthy and cobblestone-like areas were calculated per cm using ImageJ software (NIH, Bethesda, MD, USA).

### Isolation and Culture of Mesenteric Lymph Node Cells

Mesenteric lymph node cells were removed aseptically at the time of sacrifice, and cells were gently dispersed through a 70-µm cell strainer to obtain single-cell suspensions. Note that 1 × 10^6^ resulting cells were cultured in RPMI-1640 with 10% FBS and 1% P/S for 72 h in the presence of 1-µg/mL anti-CD3/CD28 monoclonal Ab, as previously described (7). For measurement of *de novo* IL-17 protein in cell supernatants, MLN cells were placed in a culture medium supplemented with 1-ng/mL TGF-β1, 20-ng/mL IL-6, and 20 U/mL IL-2 for 72 h, and then stimulated with 50-ng/mL PMA, 1-µg/mL ionomycin, and 1 × GolgiStop for 4 h at 37°C (25). After the incubation period, the cells were collected for flow-cytometry assay, as described below, and supernatants were collected for IL-10, IL-13, IL-17, TNF-α, and IFN-γ analysis by ELISA, according to the manufacturer’s instructions.

### Isolation of Lamina Propria Mononuclear Cells

Ilea were collected from experimental mice, rinsed in ice-cold PBS, and cut into pieces of approximately 0.5 cm. To remove epithelial cells and intraepithelial lymphocytes, tissues were placed in 25-mL Ca^2+^- and Mg^2+^-free HBSS supplemented with 5-mM EDTA and 1-mM DTT, and shaken for 30 min at 250 rpm at 37°C. The remaining tissues were finely fragmented, placed in 30-mL RPMI medium supplemented with 10% FBS, 0.8-µg/mL dispase and 0.1-µg/mL collagenase D, and digested for 1 h at 37°C. Cells were collected by centrifugation at 1,300 rpm for 5 min at room temperature (RT). Cell pellets were then analyzed by flow cytometry.

### Quantitative Real-time RT-PCR

Total RNA was isolated from homogenized ileal tissues using the RNeasy Mini kit (Qiagen, Valencia, CA, USA). cDNA was generated from 1 µg of RNA with maize mosaic virus random hexamers (Invitrogen). Semi-quantification of the target genes was carried out by real-time RT-PCR using SYBR Green methodology. Relative expression of each target gene was calculated by the ΔΔCt method (44). The expression of FoxP3, IL-17A, and β-actin mRNA was evaluated by using the following primer sequences: FoxP3, (5′-CCCAGGAAAGACAGCAACCTT-3′ and 5′-TTCTCACAACCAGGCCACTTG-3′); IL-17A (5′-TTTAACTCCCTTGGCGCAAAA-3′ and 5′-CTTTCCCTCCGCATTGACAC-3′); β-actin (5′-CAGGGTGTGATGGTGGGAATG-3′ and 5′-GTAGAAGGTGTGGTGCCAGATC-3′).

### Flow Cytometry

To identify T_regs_, freshly isolated LP or cultured MLN cells were stained with a mouse T_reg_ staining kit, according to the manufacturer’s instructions. Briefly, lymphocytes were blocked for 10 min on ice with anti-mouse CD16/CD32 Abs, and then stained with anti-mouse CD4 and CD25 Abs, for 30 min at 4°C in the dark. After washing, cells were stained with live/dead Fixable Violet Dead Cell Stain Kit (Thermo Scientific, Waltham, WA, USA) to determine cell viability, followed by incubation with a fixation/permeabilization buffer (eBioscience) for 30 min at 4°C in the dark. Cells were then washed with permeabilization buffer and stained with anti-mouse FoxP3 and IL-17A Abs for 30 min at 4°C in the dark. To collect live ILCs from MLNs, viability stain was used as indicated above. Cells were then stained with a combination of fluorescently conjugated monoclonal Abs optimized in a previous work (45) for 30 min at 4°C or at RT, to detect cell surface and intracellular proteins, respectively. Flow-cytometric acquisition was performed on a BD FACS LSR II instrument for T_regs_, and on a FACSAria sorter for ILCs. Data were subsequently analyzed using FlowJo_V10 software (Tree Star) by gating on live cells based on forward vs. side scatter profiles, then gating on singlets using forward scatter area vs. height, followed by dead-cell exclusion and then cell subset-specific gating (Figures S2 and S3 in Supplementary Material). CountBright™ absolute counting beads (Thermo Scientific) were used to determine absolute cell number of ILCs by flow cytometry, according to the manufacturer’s instructions.

### Statistical Analysis

Data reported in the current work are representative of three independent experiments. For comparisons made between any given two groups with normal or not normal distribution, Student’s *t*-test (two-tailed) or Mann–Whitney test was used, respectively. Provided the data fulfilled the assumptions for parametric statistics, comparison between more than two groups was carried out by two-way ANOVA with Bonferroni’s *post hoc* test. All data were expressed as median ± interquartile range with ≥95% confidence intervals. An alpha level of 0.05 was considered significant. All statistical analyses were performed using GraphPad Prism (version 7.03; GraphPad Software, San Diego, CA, USA).

## Results

### Death Receptor 3 (DR3) Stimulation Exacerbates Ileitis in SAMP Mice

Converging animal studies have shown that treatment with 4C12 prevented the development of allergic lung inflammation (26), promoted the survival of cardiac allografts (27), and reduced graft vs. host disease (28), through the expansion of T_regs_. Hence, we sought to test whether 4C12 treatment could delay or even ablate disease onset in a well-characterized mouse model of CD-like ileitis, i.e., SAMP1/YitFc (SAMP). SAMP mice spontaneously develop chronic ileitis that resembles human CD, characterized by severe inflammation in the terminal ileum, skip lesions, transmural inflammation, granulomas, crypt hyperplasia, infiltration of both acute and chronic inflammatory cells, spontaneous skin lesions, and in some instances perianal fistulas (46–48).

**Figure 1:**
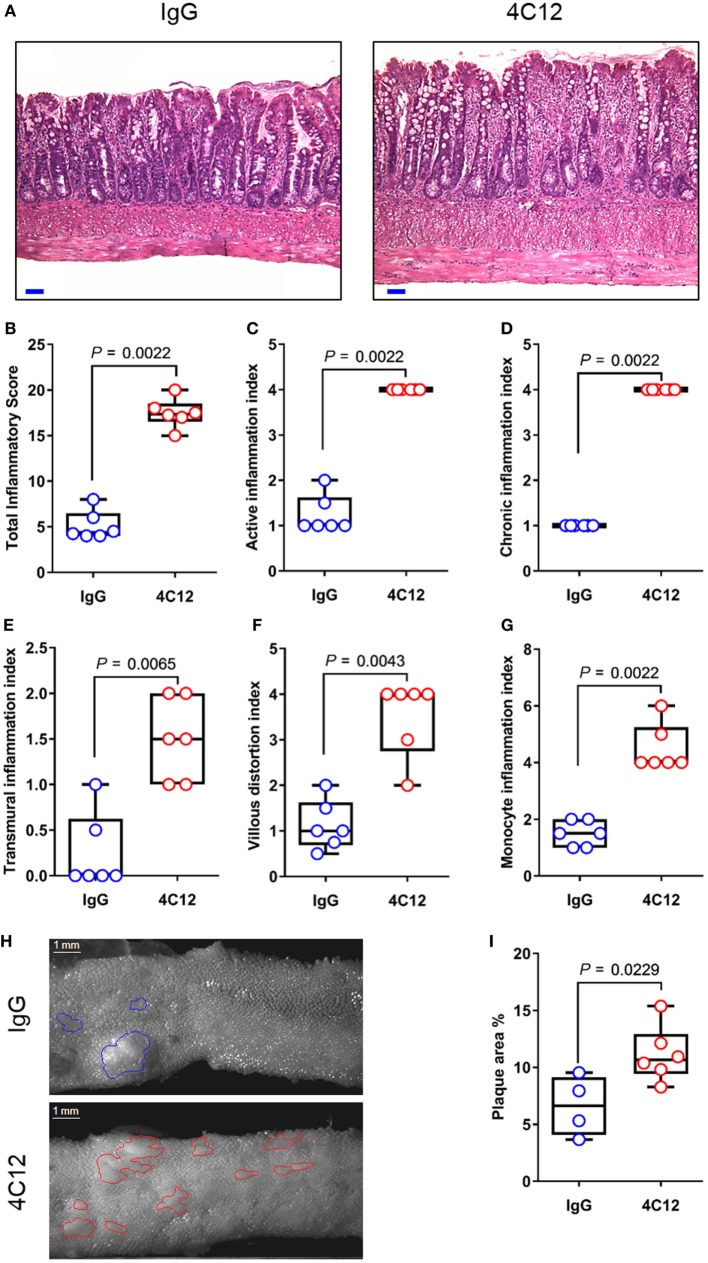
DR3 stimulation accelerates ileitis development in SAMP mice. **(A)** Representative photomicrographs of ileal sections of SAMP mice treated with control IgG isotype (left panel) or with 4C12 (right panel). Scale bar is 50 µm. **(B)** Total histologic score represents the sum of five indices including **(C)** active inflammation, **(D)** chronic inflammation, **(E)** transmural inflammation, **(F)** villous distortion, and **(G)** monocyte inflammation. Data presented as median ± interquartile range and analyzed by Mann–Whitney test, *n* = 6. **(H)** Mucosal architecture of fixed postmortem ileal specimens collected from 4C12-treated and IgG-treated SAMP mice was examined by stereomicroscopy. **(I)** Cobblestone area expressed as percentage of total specimen calculated in the ileum of 4C12-treated and IgG-treated SAMP mice. Data presented as median ± interquartile range and analyzed by two-tailed unpaired *t*-test, *n* = 4–6. Data are representative of three independent experiments.

In this study, we administered 4C12 or control Ab weekly to SAMP mice prior to disease manifestation (5-week-old), along with AKR littermates. After 4 weeks, 4C12-treated SAMP mice exhibited significantly increased ileitis severity (3.7-fold) compared with IgG-treated mice (17.7 ± 1.6 vs. 4.8 ± 1.6, *P* = 0.0022, Figures 1A,B). SAMP mice that received 4C12 were characterized by higher inflammation scores compared with controls, including 3.4-fold increase in active inflammation index (4.0 ± 0.0 vs. 1.2 ± 0.4, *P* = 0.0022, Figure 1C), fourfold increase in chronic inflammation index (4.0 ± 0.0 vs. 1.0 ± 0.0, *P* = 0.0022, Figure 1D), and fivefold increase in transmural inflammation index (1.7 ± 0.5 vs. 0.3 ± 0.5, *P* = 0.0065, Figure 1E). Additionally, distorted villous architecture, such as broadening and blunting (3.5-fold increase, 3.5 ± 0.8 vs. 1.0 ± 0.0, *P* = 0.0022, Figure 1F), and inflammatory infiltrates in the LP (3.4-fold increase, 4.5 ± 0.8 vs. 1.3 ± 0.5, *P* = 0.0022, Figure 1G) were significantly elevated in 4C12-treated mice compared with controls. Using an established protocol (43), we performed stereomicroscopic 3D-pattern profiling analysis of ileal tissue, revealing that 4C12-treated SAMP mice harbored a higher number and wider area of cobblestone lesions per cm, compared with controls (1.7-fold increase, 11.2 ± 2.4 vs. 6.6 ± 2.6, *P* = 0.0229, Figures 1H,I). Stimulation of DR3 in AKR mice did not have any effects on the health of the rodents (Figure S1 in Supplementary Material). These findings suggest that activation of DR3 signaling prior to disease manifestation accelerates inflammation occurrence, worsening ileitis development in a susceptible host.

### DR3 Stimulation Increases FoxP3^+^ Regulatory T-Cell Population but Has No Effects on T-Helper Type-17 (T_H_17) Subset

**Figure 2:**
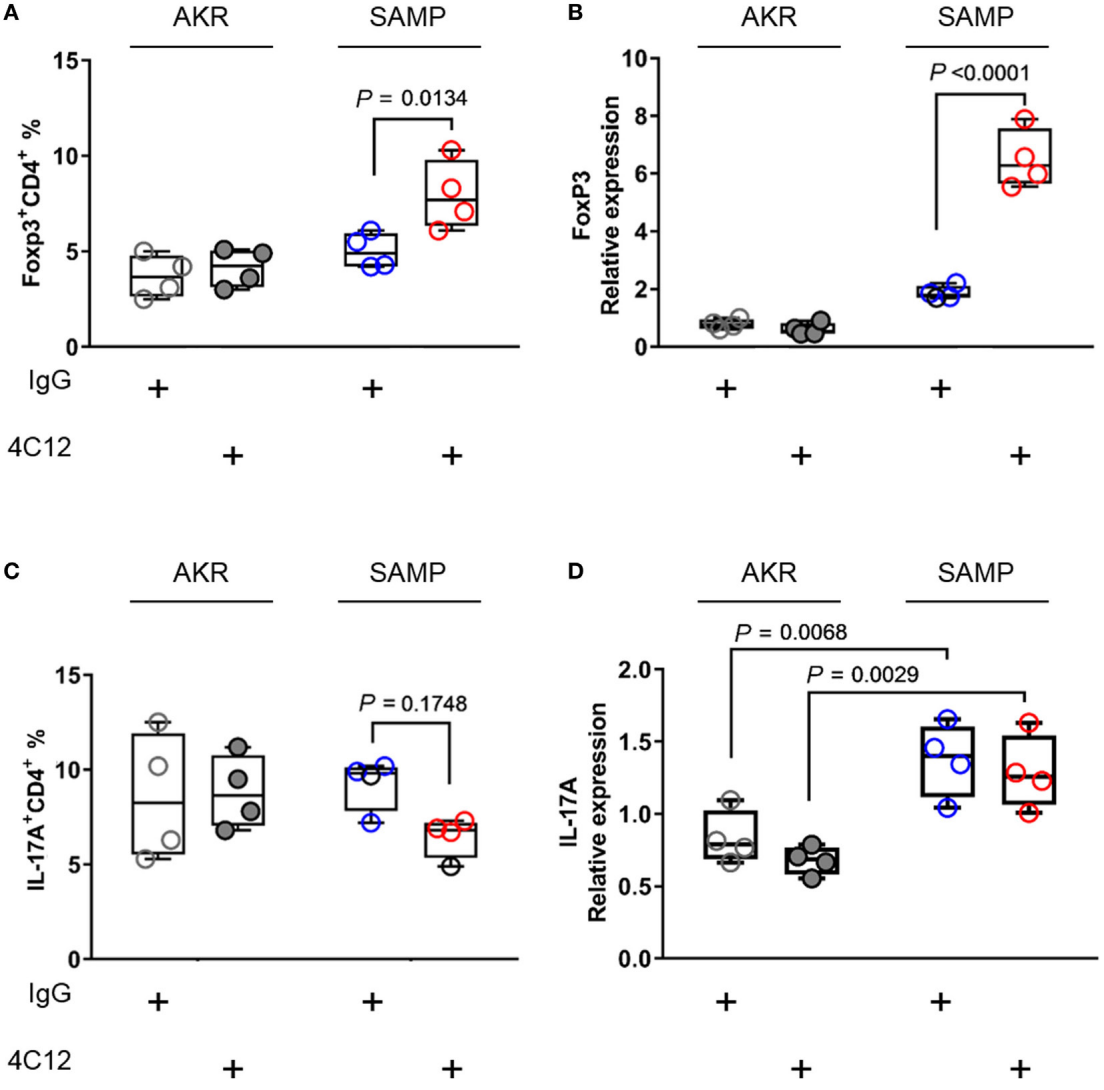
DR3 stimulation increases FoxP3^+^ T_regs_ without altering T_H_17 cell population during chronic ileitis. **(A)** Flow-cytometric analysis of mesenteric lymph node cells from IgG- and 4C12-treated SAMP or AKR mice after staining with specific anti-CD4 and anti-FoxP3 Abs or **(C)** anti-CD4 and anti-IL17 Abs. **(B)** Relative expression of FoxP3 mRNA and **(D)** of IL-17A mRNA was measured in total tissue RNA extracted from the terminal ilea of SAMP or AKR mice treated with IgG or 4C12 (10-week-old, *n* = 4). The relative expression of each target gene was normalized to the relative expression of β-actin in the sample. Data presented as median ± interquartile range and analyzed by two-way ANOVA, with Bonferroni’s *post hoc* test. Data are representative of three independent experiments.

Stimulation of DR3 with 4C12 leads to the expansion of *bona fide* CD4^+^FoxP3^+^T_regs_ (FoxP3^+^ T_regs_), which are able to dampen host autoaggression in health (26–28). In addition to modulating this T-cell subpopulation, activation of DR3 pathway may trigger a signaling cascade that plays a role in T_H_17 cell network (49, 50). Considering that T_reg_ and T_H_17 cells share key mediators essential for cell differentiation, such as TGF-β1 (51), we can infer that DR3 signaling may modulate the homeostasis of both subsets. Hence, first, we asked whether treatment with 4C12 could enrich FoxP3^+^ T_regs_ in SAMP mice. Second, we investigated whether the effect of DR3 activation on FoxP3^+^ T_regs_ was coupled with a modulation of the expression of IL-17 in our system. We observed a significant increase in the proportion of FoxP3^+^ T_regs_ in the MLN of 4C12-treated SAMP mice compared with those from the IgG-treated group (1.6-fold increase, 8.0 ± 1.8 vs. 5.0 ± 0.9, *P* = 0.0134, Figure 2A). Also, these data positively correlate with mRNA relative abundance of FoxP3 gene in ileal specimens (3.5-fold increase, 6.5 ± 1.0 vs. 1.9 ± 0.2, *P* < 0.0001, Figure 2B). On the other hand, administration of 4C12 did not significantly alter the frequency of CD4^+^IL-17^+^ cells (T_H_17s), although a contraction of T_H_17 frequency was observed in 4C12-treated SAMP mice compared with controls (0.7-fold decrease, 6.5 ± 1.1 vs. 9.3 ± 1.4, *P* = 0.1748, Figure 2C). IL-17 mRNA expression was significantly upregulated in the ileum of SAMP mice in comparison to that of AKR mice; however, we did not detect any differences in each mouse strain with regard to 4C12 administration (Figure 2D). Our data indicate that DR3 signaling expands FoxP3^+^ T_regs_, but not T_H_17s, in MLN and LP cells of a susceptible host, but not of a healthy individual.

### DR3 Stimulation Enriches CD25^−^FoxP3^+^ at the Expense of CD25^+^FoxP3^+^ Cells

**Figure 3:**
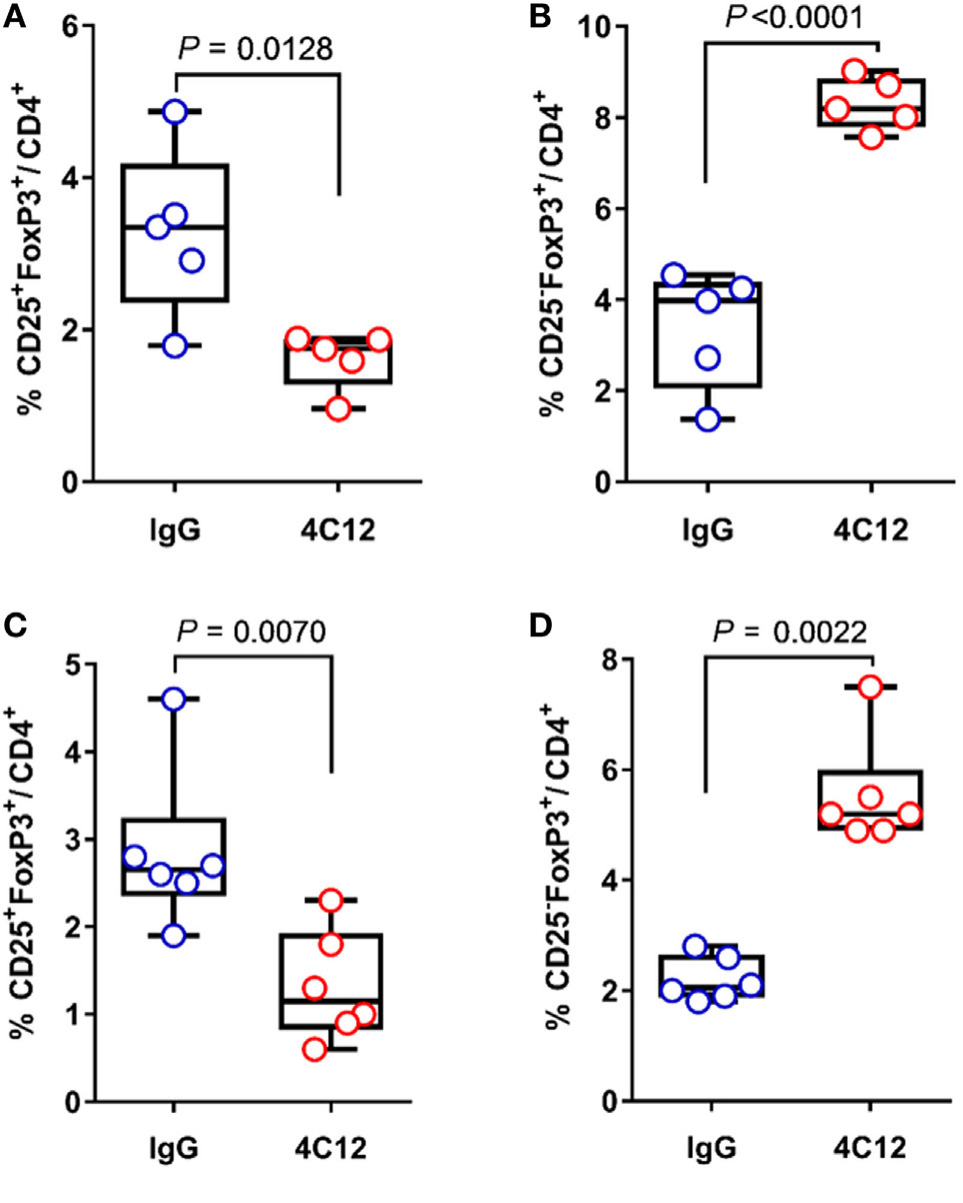
DR3 stimulation correlates to the expansion of CD25^−^FoxP3^+^ cells during chronic ileitis. **(A,B)** Flow-cytometric analysis of mesenteric lymph node and **(C,D)** lamina propria cells from IgG- and 4C12-treated SAMP mice (10-week-old, *n* = 6–5) after staining with specific anti-CD25 and anti-FoxP3 Abs. The frequency of CD25^+^FoxP3^+^ and CD25^−^FoxP3^+^ cells is indicated. All data are presented as median ± interquartile range. Data in graphs A, B, and C were analyzed by two-tailed unpaired *t*-test. Data in graph D were analyzed by Mann–Whitney test. Data are representative of three independent experiments.

Despite having FoxP3^+^ T_regs_ enriched in MLN (Figure 2A) and FoxP3 mRNA upregulated in the ileum, 4C12-treated SAMP mice exhibited a more severe ileitis compared with IgG-treated controls (Figure 1). CD4^+^CD25^+^FoxP3^+^ T_regs_, which play a central role for the maintenance of immune homeostasis, are known to be generated in the thymus (thymus-derived cells or natural T_reg_) and in the periphery (peripheral-derived cells or inducible T_reg_,) (52). Of note, peripheral-derived T_regs_ include a major subset of CD4^+^CD25^−^FoxP3^+^ and a relatively small subset of CD4^+^CD25^+^FoxP3^+^ cells. CD25 is the α-chain of the IL-2 receptor (IL-2R) and it is a T-cell activation marker as well as a T_reg_ marker. It interacts with the β- and γ-chains of IL-2R, to form a high-affinity receptor, which promotes cell proliferation and functions (53). It has been shown that, when TL1A recruits DR3 on T cells, they become highly responsive to endogenous IL-2 *via* IL-2R, resulting in cell proliferation. With these premises and with the intent to identify T_reg_ subtypes, we further investigated the contribution of CD25 to the regulatory pool in our model. Our data indicated that, in SAMP mice upon 4C12 treatment, MLN and LP lymphocytes are partially depleted (twofold) of CD25^+^FoxP3^+^ cells compared with controls (1.6 ± 0.4 vs. 3.3 ± 1.1, *P* = 0.0128, Figure 3A; 1.3 ± 0.6 vs. 2.9 ± 0.9, *P* = 0.0070, Figure 3C). This phenomenon was accompanied by a concurrent enrichment (2.5-fold) of CD25^−^FoxP3^+^ cells (8.3 ± 0.6 vs. 3.4 ± 1.3, *P* < 0.0001, Figure 3B; 5.5 ± 1.0 vs. 2.2 ± 0.4, *P* = 0.0022, Figure 3D). Our results demonstrate that, in a susceptible host, DR3 activation correlates to the reduction of CD25^+^FoxP3^+^ in favor of CD25^−^FoxP3^+^ cells. To get further insights into this observation, we carried out extensive immunophenotyping of CD25^+^FoxP3^+^ and CD25^−^FoxP3^+^ cells in unmanipulated mice. Our analysis revealed that, by expressing specific markers for T_reg_ activation and suppressive functions (i.e., Nrp-1, Helios, CTLA-4, GITR, Icos, CD103, and CD73) and by producing IL-10, CD25^+^FoxP3^+^ cells may exert regulatory functions, which are instead impaired in CD25^−^FoxP3^+^ cells (Tables S1 and S2 in Supplementary Material). Interestingly, the frequency of DR3-expressing cells was significantly elevated in the CD25^+^FoxP3^+^ subset compared with CD25^−^FoxP3^+^ subpopulation, in MLNs of both AKR and SAMP mice (*p* ≤ 0.0052, Tables S1 and S2 in Supplementary Material), suggesting a higher dependency of CD25^+^FoxP3^+^ cells on DR3 signals. Taken together, these results suggest that DR3 activation in SAMP mice promotes the switch of regulatory CD25^+^FoxP3^+^ cells to a non-regulatory CD25^−^FoxP3^+^ subpopulation.

### DR3 Deficiency Ameliorating Ileitis Severity and Expanding CD25^+^FoxP3^+^ Cells

**Figure 4:**
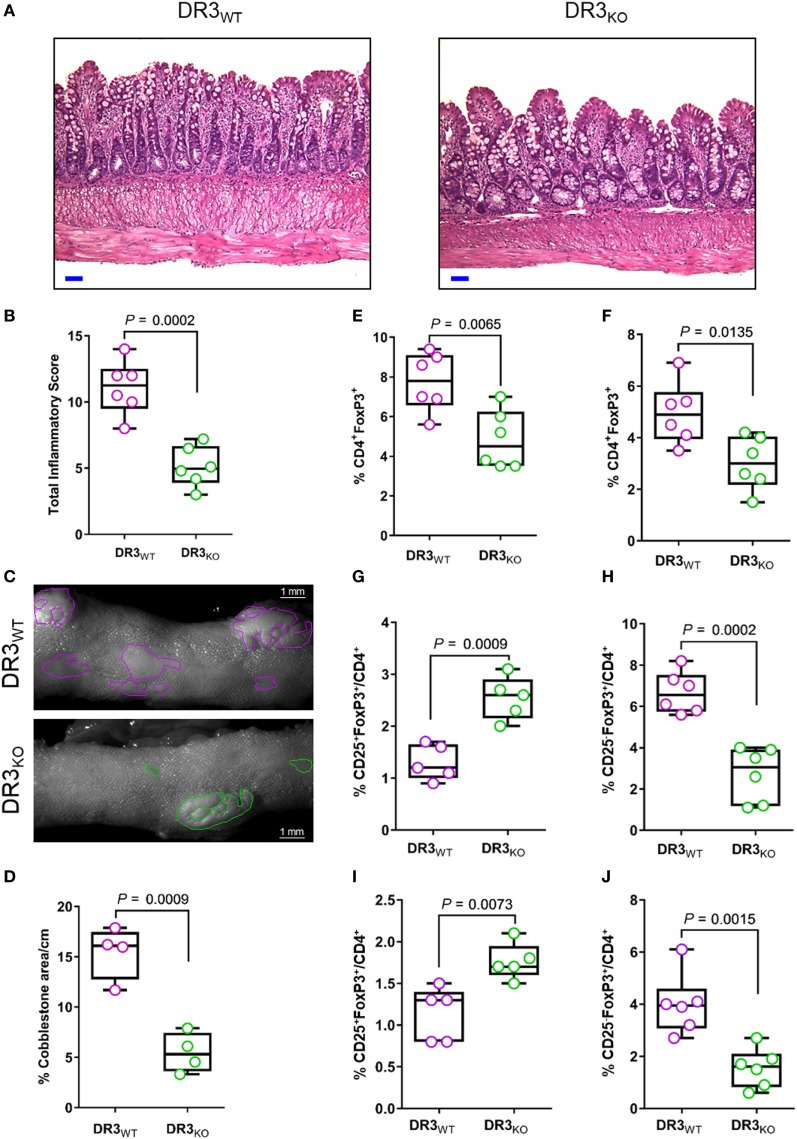
DR3 deletion ameliorates ileitis severity and expands CD25^+^FoxP3^+^ cells in SAMP mice. **(A)** Representative photomicrographs of ileal sections of SAMP mice wild-type (DR3_WT_) and lacking DR3 (DR3_KO_). Scale bar is 50 µm. **(B)** Total histologic score presented as median ± interquartile range and analyzed by two-tailed unpaired *t*-test, *n* = 6. **(C)** Fixed postmortem ileal specimens collected from DR3_WT_ and DR3_KO_ mice (10-week-old, *n* = 6) and analyzed by stereomicroscopy to assess area of abnormal (i.e., cobblestone lesions) and normal mucosa. **(D)** Cobblestone area expressed as percentage of total specimen calculated in the ileum of DR3_WT_ and DR3_KO_ mice (10-week-old, *n* = 6). Data presented as median ± interquartile range and analyzed by two-tailed unpaired *t*-test, *n* = 4. **(E)** Flow-cytometric analysis of mesenteric lymph node (MLN) and **(F)** lamina propria (LP) cells from DR3_WT_ and DR3_KO_ mice (10-week-old, *n* = 6) after staining with specific anti-CD4 and anti-FoxP3. Data presented as median ± interquartile range and analyzed by two-tailed unpaired *t*-test, *n* = 6. **(G,H)** Flow-cytometric analysis of MLN and **(I,J)** LP cells from DR3_WT_ and DR3_KO_ mice (10-week-old, *n* = 6) after staining with specific anti-CD25 and anti-FoxP3 Abs. Data presented as median ± interquartile range and analyzed by two-tailed unpaired *t*-test. Data are representative of three independent experiments.

In the current work, we demonstrated that DR3 signaling controls the balance between T_reg_ subsets favoring the enrichment of CD25^−^FoxP3^+^ over CD25^+^FoxP3^+^ cells in SAMP mice. Next, we tested whether the lack of DR3 in SAMP mice (DR3_KO_) modulated the frequency of FoxP3^+^ subpopulations in comparison to wild-type counterparts (DR3_WT_), possibly promoting CD25^+^FoxP3^+^ cell expansion. As shown in Figure 4A, the ileum of DR3_KO_ mice featured diminished villous distortion, transmural inflammation, and lymphocyte infiltration in the tissue in comparison to controls, resulting in a substantial decrease in ileitis severity in the former group (2.2-fold, 5.1 ± 1.5 vs. 11.1 ± 2.1, *P* = 0.0002, Figure 4B). The percentage of the area affected by cobblestones per cm of ileum was significantly reduced (2.8-fold) in DR3_KO_ compared with DR3_WT_ mice (5.4 ± 2.0 vs. 15.4 ± 2.6, *P* = 0.0009, Figures 4C,D). Interestingly, even though MLN and LP cells from DR3_KO_ mice presented lower distribution of FoxP3^+^ T_regs_ compared with those from DR3_WT_ mice (1.6-fold, 4.8 ± 1.5 vs. 7.8 ± 1.5, *P* = 0.0065, Figure 4E; 1.6-fold, 3.0 ± 1.0 vs. 5.0 ± 1.2, *P* = 0.0135, Figure 4F), these organs were enriched in CD25^+^FoxP3^+^ cells (twofold, 2.5 ± 0.4 vs. 1.3 ± 0.3, *P* = 0.0009, Figure 4G; 1.8 ± 0.2 vs. 1.1 ± 0.3, *P* = 0.0073, Figure 4I), at the expense of CD25^−^FoxP3^+^ cells (2.5-fold, 2.7 ± 1.3 vs. 6.7 ± 1.0, *P* = 0.0002, Figure 4H; 1.6 ± 0.7 vs. 4.0 ± 1.2, *P* = 0.0015, Figure 4J). These findings indicate that genetic deletion of DR3 improves CD-like inflammation in a susceptible host, and it is associated with the contraction of CD25^−^FoxP3^+^ in favor of the expansion of CD25^+^FoxP3^+^ cells.

### DR3 Signaling Correlates with Increased T-Helper Type-1 (T_H_1) and T-Helper Type-2 (T_H_2) Responses and Mitigates Anti-inflammatory Processes during Chronic Ileitis

**Figure 5:**
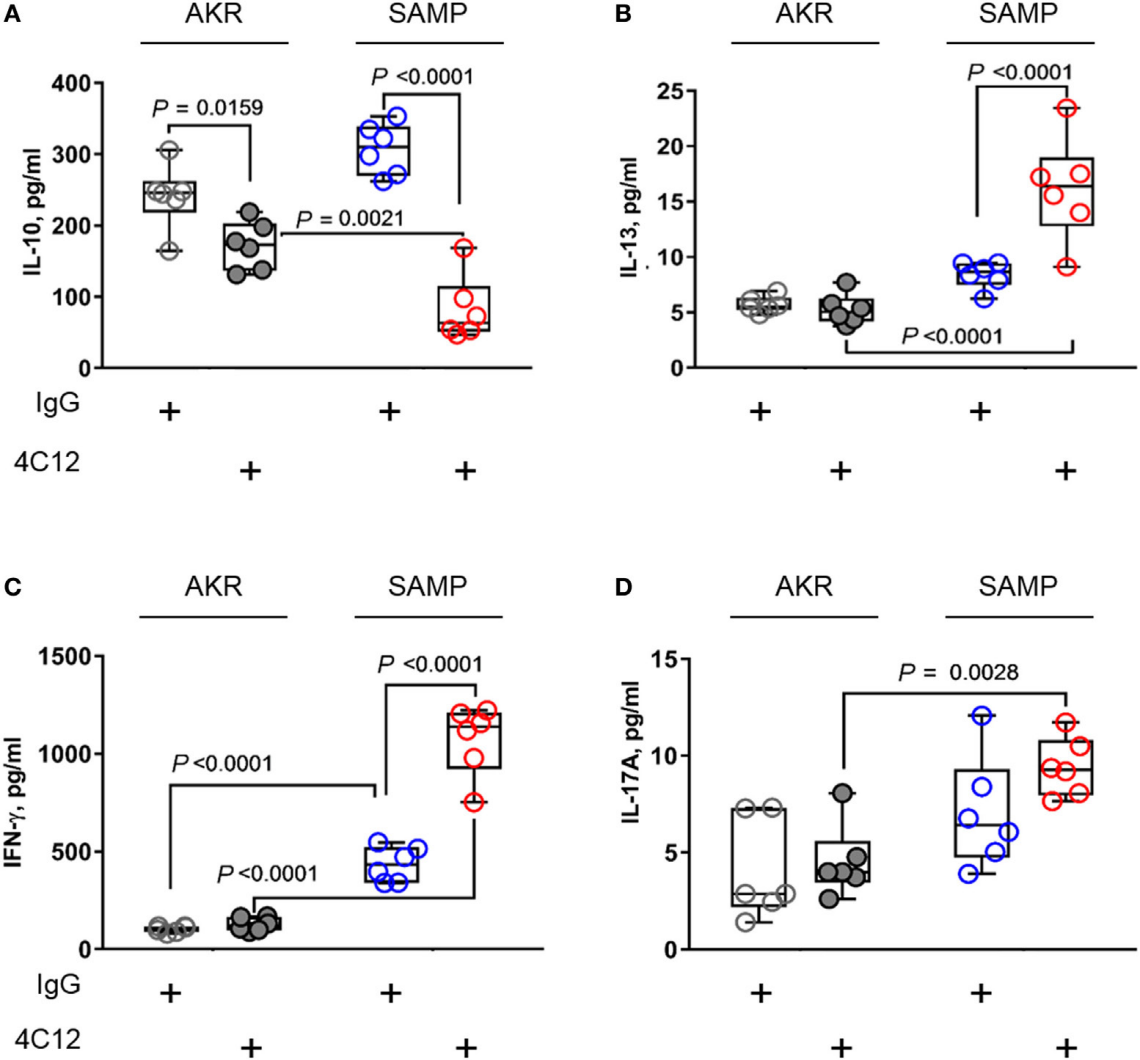
DR3 stimulation triggers T_H_1 and T_H_2 mediators and reduces anti-inflammatory response during chronic ileitis. Mesenteric lymph node cells from IgG- or 4C12-treated SAMP and AKR mice (10-week-old, *n* = 6) were cultured in RPMI medium supplemented with anti-CD3/CD28 Abs. After 72 h, the secretion of indicated cytokines was quantified in cell supernatants [IL-10 **(A)**, IL-13 **(B)**, IFN-γ **(C)**, IL-17A **(D)**]. Data presented as median ± interquartile range and analyzed by two-way ANOVA, with Bonferroni’s *post hoc* test. Data are representative of three independent experiments.

In an effort to investigate T-cell functions, we stimulated MLN cells with anti-CD3/CD28 Abs and measured protein levels secreted in cell supernatants. Lymphocytes from 4C12-treated SAMP mice produced higher levels of T_H_1 and T_H_2 cytokines compared with those from the IgG-treated group (respectively, IFN-γ, 2.5-fold increase with 1072.3 ± 179.5 vs. 435.1 ± 88.9, *P* < 0.0001, Figure 5B; IL-13, 1.9-fold increase with 16.1 ± 4.7 vs. 8.4 ± 1.2, *P* < 0.0001, Figure 5C). Additionally, TNF-α level was also increased upon DR3 stimulation (82.2 ± 46.2 vs. 307.0 ± 35.9, *P* = 0.0018, Figure S4 in Supplementary Material). In contrast, IL-10 secretion was found dramatically reduced (3.7-fold) in MLN cells from 4C12-treated SAMP mice compared with controls (82.2 ± 46.2 vs. 307.0 ± 35.9, *P* < 0.0001, Figure 5A). We observed higher secretion (1.3-fold) of IL-17A protein by MLN cells from SAMP mice compare to those from AKR, indicating that *de novo* T_H_17 response is enhanced under these experimental conditions, independently of the treatment administered here (9.4 ± 1.5 vs. 7.0 ± 2.9, *P* = 0.0028, Figure 5D).

### DR3 Deficiency is Associated with Increased Anti-inflammatory Response and Concomitant Ablation of T_H_1, T_H_2, and T_H_17 Mediators in SAMP Mice

**Figure 6:**
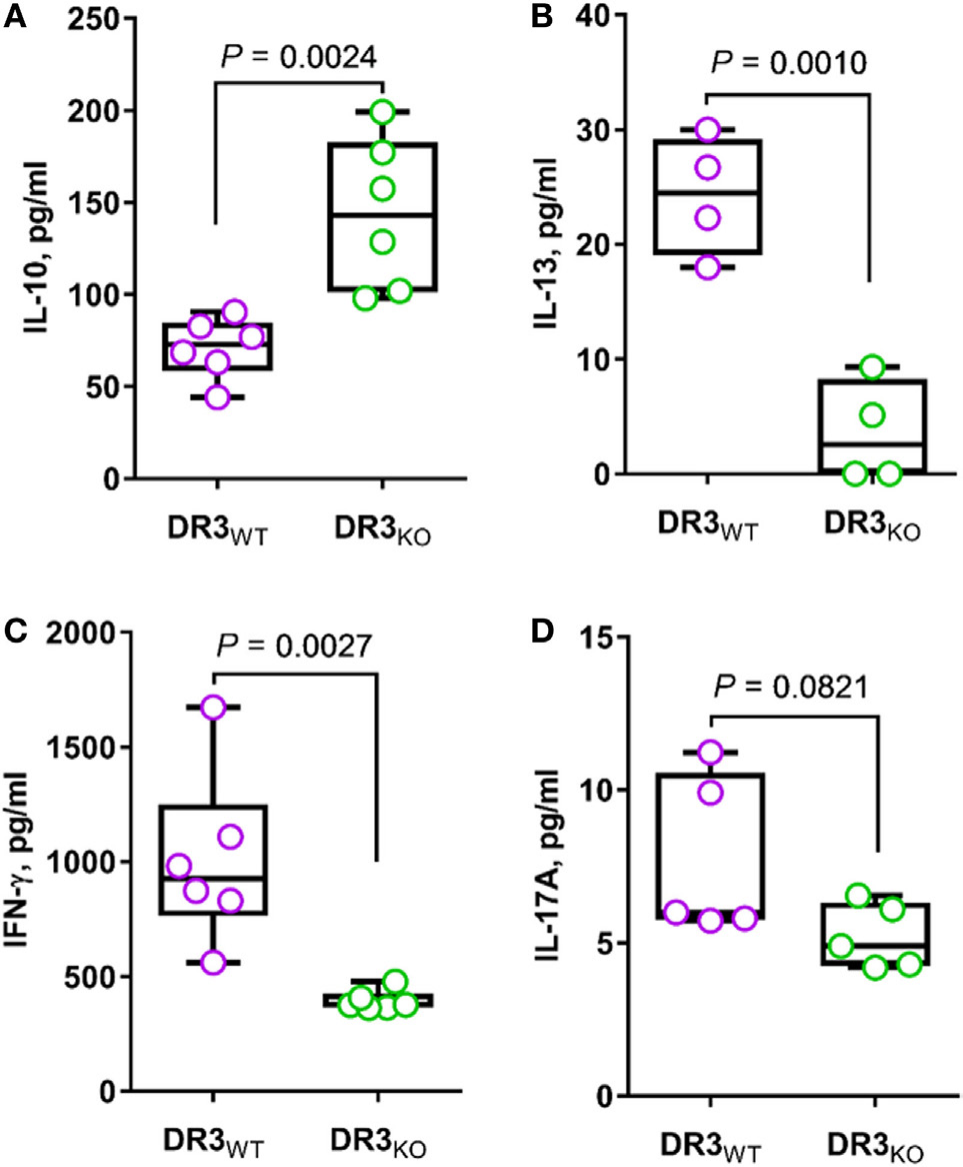
Genetic deletion of DR3 enhances anti-inflammatory response and reduces T_H_1, T_H_2, and T_H_17 cytokines in SAMP mice. Mesenteric lymph node cells from DR3_WT_ and DR3_KO_ mice (10-week-old, *n* = 6) were cultured in RPMI medium supplemented with anti-CD3/CD28 Abs. After 72 h, the secretion of indicated cytokines was quantified in cell supernatants [IL-10 **(A)**, IL-13 **(B)**, IFN-γ **(C)**, IL-17A **(D)**]. Data presented as median ± interquartile range and analyzed by two-tailed unpaired *t*-test. Data are representative of three independent experiments.

With the intent of assessing the contribution of DR3 to T-cell function, we stimulated MLN cells from DR3-deficient SAMP (DR3_KO_) mice with anti-CD3/CD28 Abs revealing a significant reduction of T_H_1, T_H_2, and T_H_17 cytokines, including IFN-γ (2.5-fold increase, 1004.3 ± 374.8 vs. 394.5 ± 44.0, *P* = 0.0027, Figure 6C), IL-13 (6.7-fold increase, 24.3 ± 5.2 vs. 3.6 ± 4.5, *P* = 0.0010, Figure 6B), and IL-17A (1.5-fold increase, 7.7 ± 2.6 vs. 5.2 ± 1.1, *P* = 0.0821, Figure 6D), compared with those from wild-type counterparts (DR3_WT_). In contrast, MLN cells from DR3_KO_ mice secreted higher IL-10 protein level than those from littermate controls (2.0-fold increase, 143.7 ± 41.1 vs. 71.0 ± 16.4, *P* = 0.0024, Figure 6A). Taken together, these results suggest that the activity of DR3 signaling is mainly pro-inflammatory in a susceptible host.

### DR3 Signaling Expands Innate Lymphoid Cell Group 1 (ILC1s) and Reduces ILC3s during Chronic Ileitis

**Figure 7:**
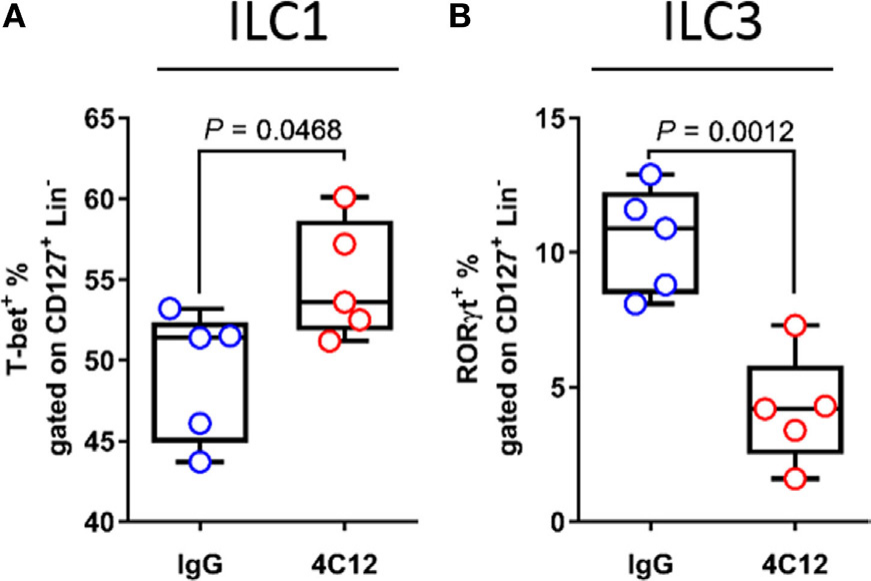
Upon death receptor 3 stimulation, increased innate lymphoid cell group 1 (ILC1) and decreased ILC3 frequencies are associated with intestinal inflammation. Flow-cytometric analysis of mesenteric lymph node cells from IgG- or 4C12-treated SAMP mice (10-week-old, *n* = 5) after staining with specific Abs for detection of ILC populations, including **(A)** T-bet^+^ ILC1s and **(B)** receptor-related orphan receptor-γt (ROR-γt^+^) ILC3s. Cell frequencies presented as median ± interquartile range and analyzed by Mann–Whitney test. Data are representative of three independent experiments.

Several reports have recently shown that DR3 is expressed on ILCs, inferring a role of this protein in ILC functionality. Furthermore, a large amount of data obtained from both human and mouse studies indicate a role for these cells in IBD, considering that some ILC subsets have regulatory functions in the healthy intestine (8, 10, 11). Hence, we used flow cytometry to measure the distribution of ILC subsets in MLNs collected from 4C12- and IgG-treated SAMP mice. Treatment with 4C12 expanded ILC1s compared with controls (1.1-fold increase, 54.9 ± 3.7 vs. 49.2 ± 4.1, *P* = 0.0468, Figure 7A). ILC3 frequency was found reduced (2.5-fold) in MLN cells from 4C12-treated mice compared with controls (4.2 ± 2.1 vs. 10.5 ± 2.0, *P* = 0.0012, Figure 7B). In contrast, the distribution of ILC2 subset was unaltered upon treatment (Figure S5A in Supplementary Material). Interestingly, the frequency of ILC3 cells expressing DR3 was significantly higher than that of ILC1s (*p* < 0.0012, Figure S5B in Supplementary Material), suggesting a higher dependency on DR3 signals of ILC3s compared with ILC1s. Hence, these data suggest that DR3 stimulation may promote the conversion of ILC3 into ILC1 cells in SAMP mice.

Considering that lack of DR3 ameliorates ileitis in SAMP mice, we anticipated a positive change in the frequency of ILC3s in DR3_KO_ mice. Remarkably, the distribution of all ILC subtypes in MLNs from DR3_KO_ mice was equal to that in the wild-type counterparts. To further investigate this result, we went on measuring the magnitude of ILC groups in our system. We found that the absolute cell number of ILCs, along with that of CD45^+^ and CD127^+^Lin^−^ cells, was significantly decreased in MLNs from DR3_KO_ compared with DR3_WT_ mice (Figure S6 in Supplementary Material). This outcome may be ascribed to the fact that DR3_KO_ mice harbor MLN organs of smaller size compared with DR3_WT_ mice (Cominelli *et al*., unpublished data) and, therefore, they carry a lower number of cell without affecting immune cell frequency.

## Discussion

In the present study, we investigated the role of DR3 signaling in the modulation of the balance between regulatory and effector lymphocytes during chronic experimental ileitis. To this end, we exploited a well-characterized mouse model for CD, i.e., SAMP1/YitFc (SAMP), in which the main pathology is a spontaneous ileitis that is strikingly similar to human CD, with skip lesions, transmural inflammation, and scarring that can lead to stricture formation (47, 48). The current understanding is that, upon TL1A binding, DR3 triggers a signaling cascade that increases the sensitivity of T cells to endogenous IL-2 *via* the IL-2 receptor (IL-2R), and enhances T-cell proliferation at the site of inflammation (54). Consistently with patients who suffer from CD, high expression levels of DR3 found in SAMP mice during the chronic phase of the disease support the concept that the TL1A/DR3 system contributes to pathogenic inflammation in this model (5–7). In line with this finding, our recent studies demonstrated that DR3 deletion in SAMP mice restores the mucosal immunostat, normalizes intestinal inflammatory gene expression, and prevents the development of inflammation-induced intestinal fibrosis, thereby affecting the functions of effector lymphocytes and their capacity to adoptively transfer ileitis (Cominelli *et al*., unpublished data). To help unraveling the complexity of the TL1A/DR3 signaling pathway in the course of IBD, in the present study, we demonstrated that DR3 deletion ameliorates disease development by promoting anti-inflammatory processes, which were associated with enrichment of CD25^+^FoxP3^+^ cells in SAMP mice. Taken together, these findings suggest that the DR3 is required on T cells for local effector T-cell expansion and effector responses in a susceptible host.

A recent innovative approach developed by Podack et al. provided proof of concept for a regulatory role of DR3 in an allergic disease model (26). With the administration of a single injection of a stimulating DR3 antibody (4C12) to immunocompetent mice, Podack et al. demonstrated its highly efficacy at reducing pathology when used prophylactically (26). Other research groups applied successfully this methodology reporting improved organ allograft survival after treatment, attributable to a systemic over-proliferation of pre-existing CD25^+^FoxP3^+^ cells *in vivo* (27, 28). Therefore, the knowledge that activation of DR3 signaling modulates T_reg_ expansion in a healthy host leads to the appealing idea of using this mechanism as a potential therapeutic target in human inflammatory disease, such as CD. Promising findings recently gathered in CD patients treated with T_regs_ have reinvigorated the enthusiasm for this therapeutic approach (55–57). Therefore, we postulated the possibility of delaying inflammation or eradicating ileitis in SAMP mice by promoting T_reg_ proliferation prior to disease manifestation. This hypothesis was addressed in the current study through the administration of 4C12 to SAMP mice prior to disease initiation. Our results indicated that, in a susceptible host, DR3 stimulation increased the frequency of Foxp3^+^ T_regs_; however, contrary to expectations, this phenomenon was associated with significant exacerbated ileitis.

When the contribution of CD25 to the T_reg_ pool was investigated in SAMP mice, it became apparent that DR3 stimulation induced the reduction of CD25^+^FoxP3^+^ cells, in favor of CD25^−^FoxP3^+^ cells with increased T_H_1/T_H_2 responses. Although it is known that CD25^−^FoxP3^+^ cells contain some regulatory activity (58–60), there is a wide consensus on the fact that adequate suppression functions require expression of CD25 (61). This finding is in agreement with previous results from our laboratory, which identified the same CD25^−^FoxP3^+^ subtype in SAMP mice upon anti-CD25 Ab treatment as dysfunctional T_regs_ overrepresented during spontaneous ileitis, which acquired a T_H_1/T_H_2 effector phenotype (60). Studies in human systemic lupus erythematosus have confirmed these findings by reporting increased CD25^−^FoxP3^+^ cells with T_H_1/T_H_2 effector phenotype (62–65). Some evidence have demonstrated that T_regs_ may exert a milieu-dependent plasticity by readily switching to an effector phenotype in inflamed sites, accelerating inflammatory processes, thereby aggravating the underlying disease (66–68). Disease pathogenesis in SAMP mice is the result of a dual mechanism of inflammation consisting in an early inductive phase (4–7 weeks of age), driven by T_H_1 responses, and a later chronic inflammatory phase (9–16 weeks of age), primarily mediated by T_H_2 effector pathways (48). In this model, intestinal inflammation can be histologically assessed around 10 weeks of age, following CD-like lesions development (43, 47). Nonetheless, similar to human CD patients in preclinical phase, even though younger mice do not show histological features of disease, inflammatory processes have already initiated in the inductive phase (48). Hence, in the current work, administration of 4C12 to SAMP mice occurred in this phase, and not prophylactically. Earlier studies showed that 4C12 treatment during active disease aggravated the pathology (69), whereas the same antibody administered prophylactically to a healthy individual protected the host from future insults (26). Of note, the intense T_H_1 responses typical of the inductive phase give rise to an inflammatory milieu (48), even before ileal tissue manifestations, that may support the dynamic conversion of regulatory cells into effector FoxP3^+^ cells, which accelerate disease development. Moreover, mice lacking functional peripheral-derived T_regs_ develop T_H_2 pathologies in the intestine and lungs, and dysbiosis (70). Additionally, extensive immunophenotyping of CD25^+^FoxP3^+^ and CD25^−^FoxP3^+^ cells in unmanipulated mice revealed that the former expresses specific markers considered to be critical for T_reg_ immunoregulatory properties, that instead are missing in CD25^−^FoxP3^+^ cells (Tables S1 and S2 in Supplementary Material). For instance, the frequency of cells expressing markers correlated to T_reg_ suppressive activity and stability, such as Nrp-1 and Helios, was dramatically reduced in the CD25^−^FoxP3^+^ subset, suggesting that this is an unstable and non-regulatory population. Moreover, the expression of DR3 was increased on CD25^+^FoxP3^+^ cells, suggesting a higher dependency of this subset on DR3 signals. Therefore, considering collectively all these data, we propose that, upon inflammatory environmental cues, DR3 stimulation targets FoxP3^+^cells and converts regulatory cells to CD25^−^FoxP3^+^cells, which are dysfunctional lymphocytes that secrete effector mediators and accelerate disease manifestations in SAMP mice. Conversely, it cannot be excluded that the apparently opposite dynamics of CD25^+^FoxP3^+^ and CD25^−^FoxP3^+^ cells may not necessarily imply a conversion from the former to the latter. In fact, based on the evidence reported by some human and mouse studies, another hypothesis may be that CD25^−^FoxP3^+^ cells are indeed activated effector T cells, which transiently upregulate FoxP3, without exerting any regulatory activity (71, 72). Finally, we propose an alternative view consisting in the possibility that CD25^−^FoxP3^+^ cells may be T_regs_ which have transiently downregulated CD25 due to the local inflammatory milieu (59, 73). Hence, future work will be focused on dissecting the functional role of CD25^+^FoxP3^+^ and CD25^−^FoxP3^+^ cells in intestinal immunity-microbiota interactions, and in controlling adaptive immunity to restrain inflammation at mucosal surfaces. Additionally, considering the interesting approach suggested by Rouse group in collaboration with Podack, where 4C12 was combined with galectin-9, a protein able to selectively inhibit effector T-cell functions during chronic stromal keratitis (74), future experiments will investigate the efficacy of this combination therapy on the balance between regulatory and effector lymphocyte in our system.

Another novel finding described here is that DR3 stimulation in SAMP mice expanded ILC1s, which produce IFN-γ, at the expense of ILC3s. In agreement with our data, the frequency of the ILC1 subset was found elevated in inflamed intestine of CD patients, underlying a role for these cells in the pathogenesis of gut mucosal inflammation (32, 37, 38). Hence, the skewed frequencies of ILCs in MLNs of 4C12-treated SAMP mice compared with controls could be explained by specific recruitment of cells to the periphery, according to environmental cues. Based on the remarkable plasticity of ILCs, a more intriguing explanation argues that ILC3s may switch to ILC1s in 4C12-treated SAMP mice. Data collected from human and mouse studies revealed that a fraction of ILC3s can downregulate ROR-γt, lose the ability to produce IL-22, and acquire the capacity to secrete IFN-γ in response to IL-12, diverting to an ILC1 phenotype (32, 75). Moreover, we showed in the current study that ILC3s from SAMP mice expressed higher levels of DR3, suggesting that this subset may be more sensitive and may represent a preferential target following DR3 manipulation. Of note, we previously demonstrated that TL1A synergizes with IL-12 to promote IFN-γ production by murine lymphocytes in SAMP mice (7). Therefore, we can infer that DR3 stimulation with 4C12 may generate a similar signaling cascade, resulting in this phenotype switch.

In conclusion, DR3 stimulation in SAMP mice aggravated the severity of ileitis possibly due to the expansion of dysfunctional CD25^−^FoxP3^+^ cells and ILC1s, both expressing an effector phenotype. The functional role of these cell subtypes during chronic inflammation needs to be further investigated in our mouse model to give better insights into the functional balance between protective and inflammatory lymphocytes. Altogether, our data suggest a model in which modulation of DR3 signaling in T_regs_, T effectors and ILCs converge in a regulatory network that controls disease development and progression. Finally, dissecting the cellular mechanisms that govern lymphocyte functions following DR3 engagement and manipulating the resulting signaling cascade may provide a novel targeted therapy for CD.

## Ethics Statement

All experimental procedures were approved by the Institutional Animal Care and Use Committee of CWRU and were in accordance with the Association for Assessment and Accreditation of Laboratory Animal Care guidelines.

## Author Contributions

ZL and LB shared the first authorship. FC contributed to the design of the study. ZL, LB, ML, L-GJ, and JDW performed the experiments. LB and ZL analyzed the data. LB and ZL drafted the manuscript. K-AB conducted ILC isolation and immunophenotyping. FC, TP, and ML analyzed and interpreted the data. All authors approved the final version of the manuscript and agreed to be accountable to all aspects of this work.

## Conflict of Interest Statement

The authors declare that the research was conducted in the absence of any commercial or financial relationships that could be construed as a potential conflict of interest.

## Supplementary Material

Figure S1 DR3 stimulation does not affect the phenotype of AKR mice. Representative photomicrographs of ileal sections of AKR mice treated with control IgG isotype or with 4C12s. Scale bar is 50 µm. Data are representative of three independent experiments.

Figure S2 Gating strategy for detection of regulatory T-cell subsets by flow cytometry. After duplet exclusion and identification of CD4^+^ lymphocytes within mesenteric lymph node cells, T cells were further separated based on the cell surface markers CD25 and the intracellular protein FoxP3.

Figure S3 Gating strategy for detection of innate lymphoid cell (ILC) populations by flow cytometry. Gating strategy exploited to identify ILC subsets in mesenteric lymph node cells, including ILC1 (CD45^+^Lineage^−^CD127^+^Gata-3^−^ROR-γt^-^T-bet^+^), ILC2 (CD45^+^Lineage^−^CD127^+^Gata-3^+^ROR-γt^-^), and ILC3 (CD45^+^Lineage^-^CD127^+^Gata-3^−^ROR-γt^+^).

Figure S4 DR3 stimulation triggers TNF-α response in SAMP mice. Mesenteric lymph node cells were collected from IgG- and 4C12-treated SAMP or AKR mice, and stimulated with anti-CD3/CD28 antibodies for 72 h. TNF-α level was quantified in cell supernatants by ELISA. Data presented as median ± interquartile range and analyzed by two-way ANOVA, with Bonferroni’s *post hoc* test. Data are representative of three independent experiments.

Figure S5 DR3 stimulation does not alter innate lymphoid cell group 2 (ILC2s) in SAMP mice. **(A)** Flow-cytometric analysis of mesenteric lymph node (MLN) cells from IgG- or 4C12-treated SAMP mice (10-week-old, *n* = 5) after staining with specific Abs for detection of gata-3^+^ ILC2s. Cell frequency presented as median ± interquartile range and analyzed by Mann–Whitney test. **(B)** Frequency of DR3-expressing ILCs in MLN cells from SAMP mice (10-week-old, *n* = 5). Data presented as median ± interquartile range and analyzed by two-way ANOVA, with Bonferroni’s *post hoc* test. Data are representative of three independent experiments.

Figure S6 DR3 deficiency is associated with constitutive reduced innate lymphoid cell (ILC) number. Flow-cytometric analysis of mesenteric lymph node cell DR3_WT_ and DR3_KO_ mice (10-week-old, *n* = 6) after staining with specific Abs for detection of ILC populations, including T-bet^+^ ILC1, gata-3^+^ ILC2, and ROR-γt^+^ ILC3. **(A,C,E,G)** Cell frequencies and **(B,D,F,H)** absolute cell numbers presented as median ± interquartile range and analyzed by two-tailed unpaired *t*-test. Data are representative of three independent experiments.

Table S1 Characterization of CD25+/–FoxP3+ cell subsets through selected markers in MLNs of unmanipulated AKR, SAMP and DR3KO mice. The results are expressed as the mean percentage ± one SD, and analyzed by 1-way ANOVA with Bonferroni post-hoc test. The number of samples is indicated in parentheses.
