# Supplementary figures and images for: Death Receptor 3 Signaling Controls the Balance between Regulatory and Effector Lymphocytes in SAMP1/YitFc Mice with Crohn’s Disease-Like Ileitis
Source: Front Immunol. 2018 Mar 1;9:362. doi: 10.3389/fimmu.2018.00362 (PMC5837992; doi:10.3389/fimmu.2018.00362)

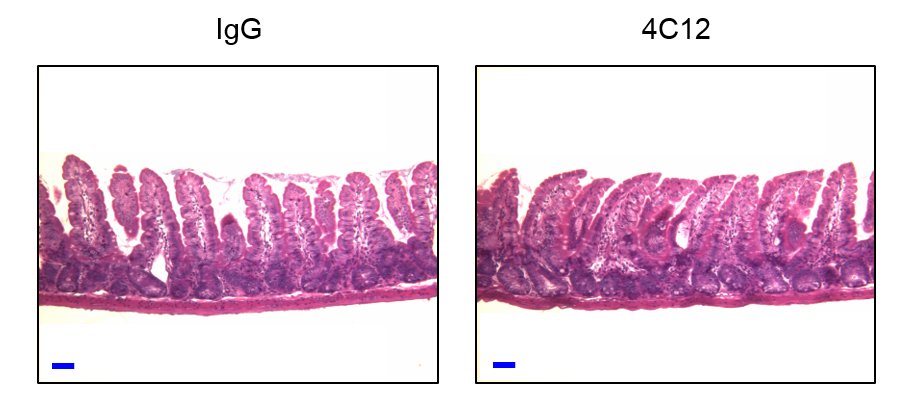

Supplement: Figure S1 — DR3 stimulation does not affect the phenotype of AKR mice. Representative photomicrographs of ileal sections of AKR mice treated with control IgG isotype or with 4C12s. Scale bar is 50 µm. Data are representative of three independent experiments. [file Image_1.TIF]

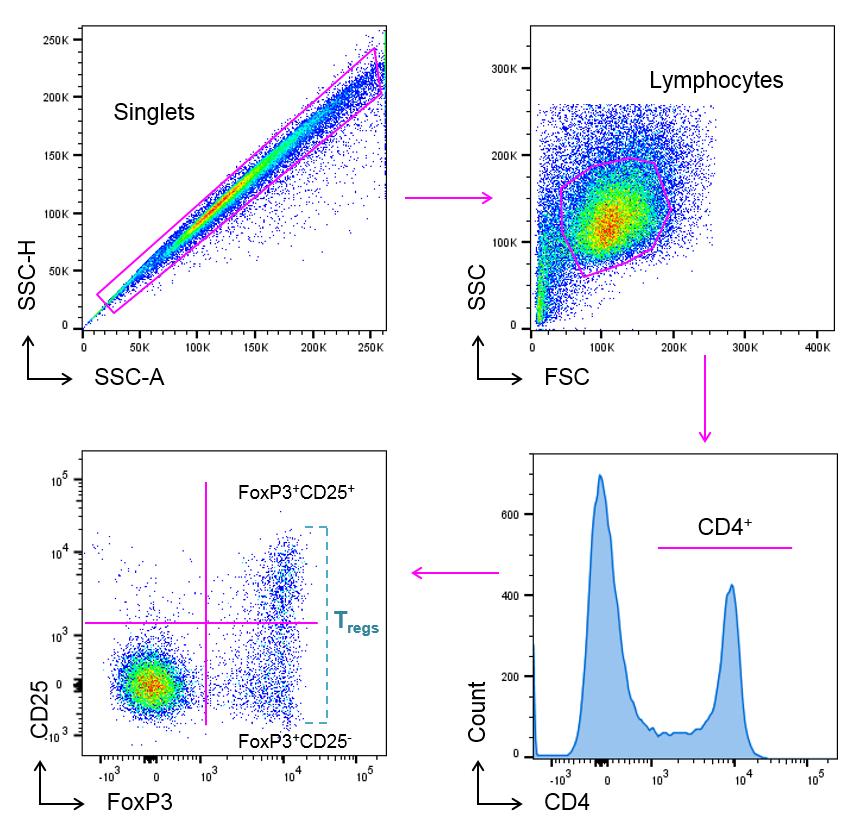

Supplement: Figure S2 — Gating strategy for detection of regulatory T-cell subsets by flow cytometry. After duplet exclusion and identification of CD4+ lymphocytes within mesenteric lymph node cells, T cells were further separated based on the cell surface markers CD25 and the intracellular protein FoxP3. [file Image_2.TIF]

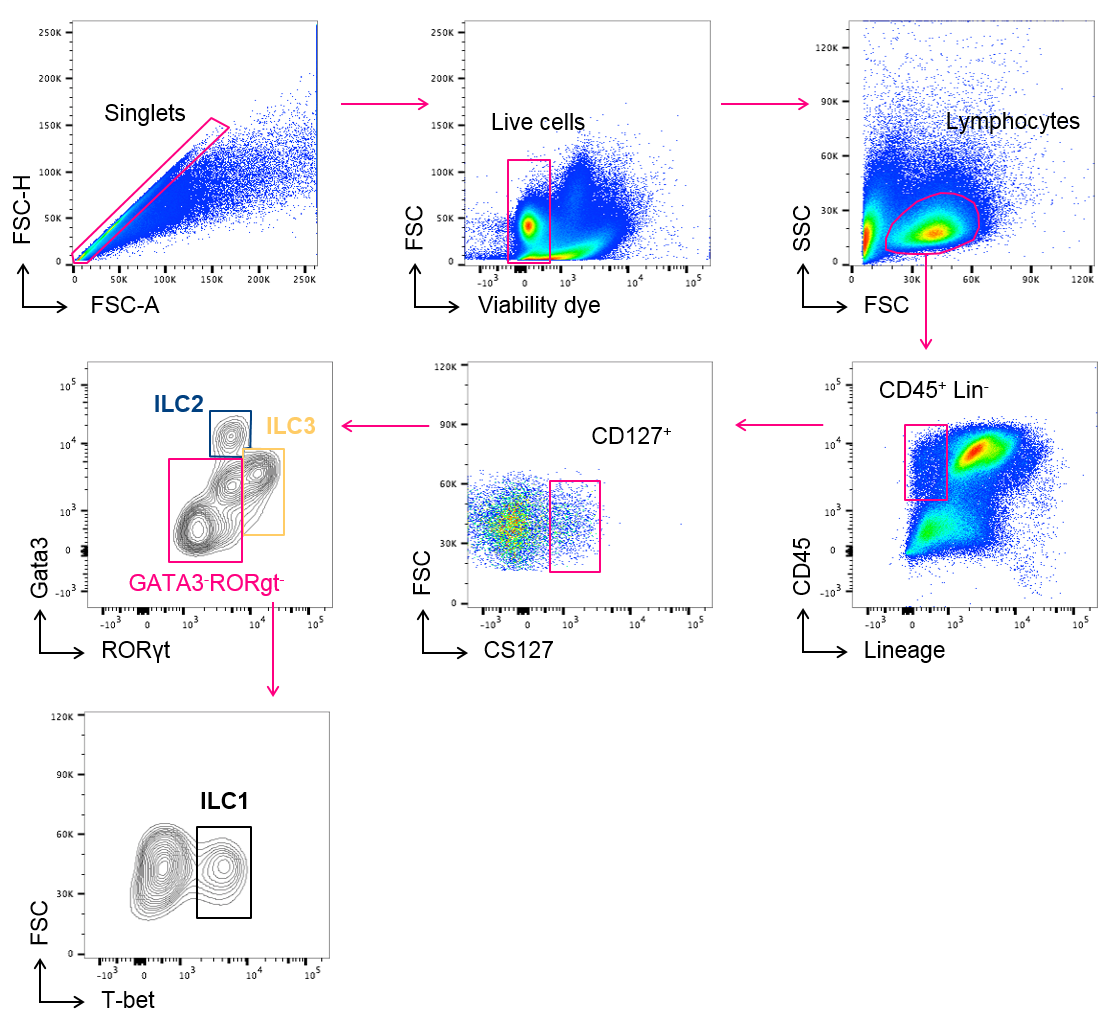

Supplement: Figure S3 — Gating strategy for detection of innate lymphoid cell (ILC) populations by flow cytometry. Gating strategy exploited to identify ILC subsets in mesenteric lymph node cells, including ILC1 (CD45+Lineage−CD127+Gata-3−ROR-γt-T-bet+), ILC2 (CD45+Lineage−CD127+Gata-3+ROR-γt-), and ILC3 (CD45+Lineage-CD127+Gata-3−ROR-γt+). [file Image_3.TIF]

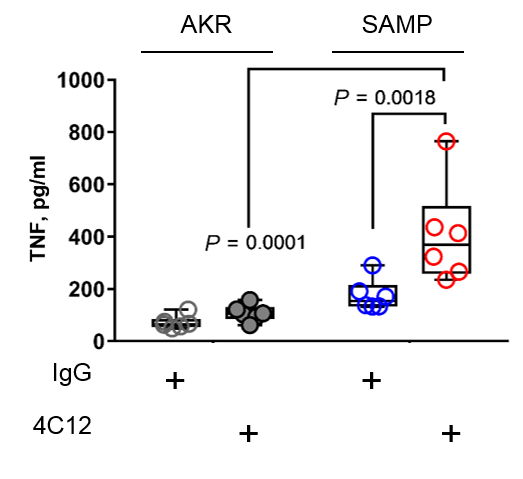

Supplement: Figure S4 — DR3 stimulation triggers TNF-α response in SAMP mice. Mesenteric lymph node cells were collected from IgG- and 4C12-treated SAMP or AKR mice, and stimulated with anti-CD3/CD28 antibodies for 72 h. TNF-α level was quantified in cell supernatants by ELISA. Data presented as median ± interquartile range and analyzed by two-way ANOVA, with Bonferroni’s post hoc test. Data are representative of three independent experiments. [file Image_4.TIF]

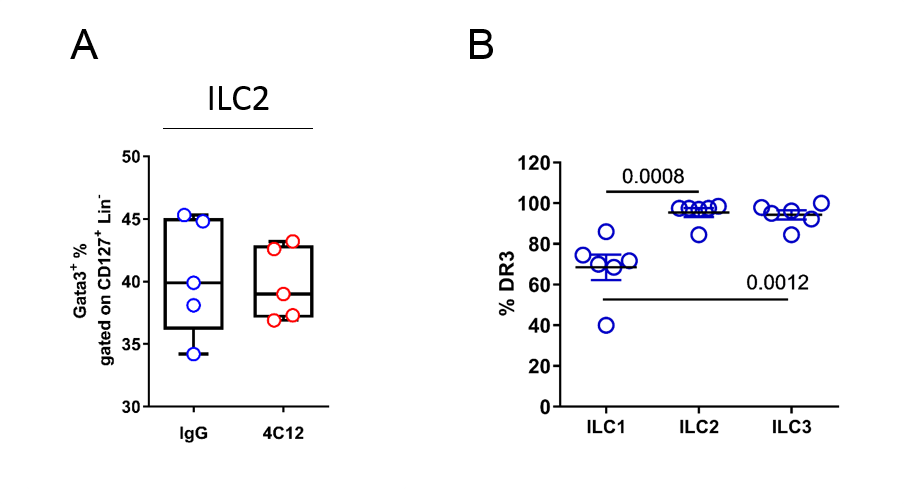

Supplement: Figure S5 — DR3 stimulation does not alter innate lymphoid cell group 2 (ILC2s) in SAMP mice. (A) Flow-cytometric analysis of mesenteric lymph node (MLN) cells from IgG- or 4C12-treated SAMP mice (10-week-old, n = 5) after staining with specific Abs for detection of gata-3+ ILC2s. Cell frequency presented as median ± interquartile range and analyzed by Mann–Whitney test. (B) Frequency of DR3-expressing ILCs in MLN cells from SAMP mice (10-week-old, n = 5). Data presented as median ± interquartile range and analyzed by two-way ANOVA, with Bonferroni’s post hoc test. Data are representative of three independent experiments. [file Image_5.TIF]

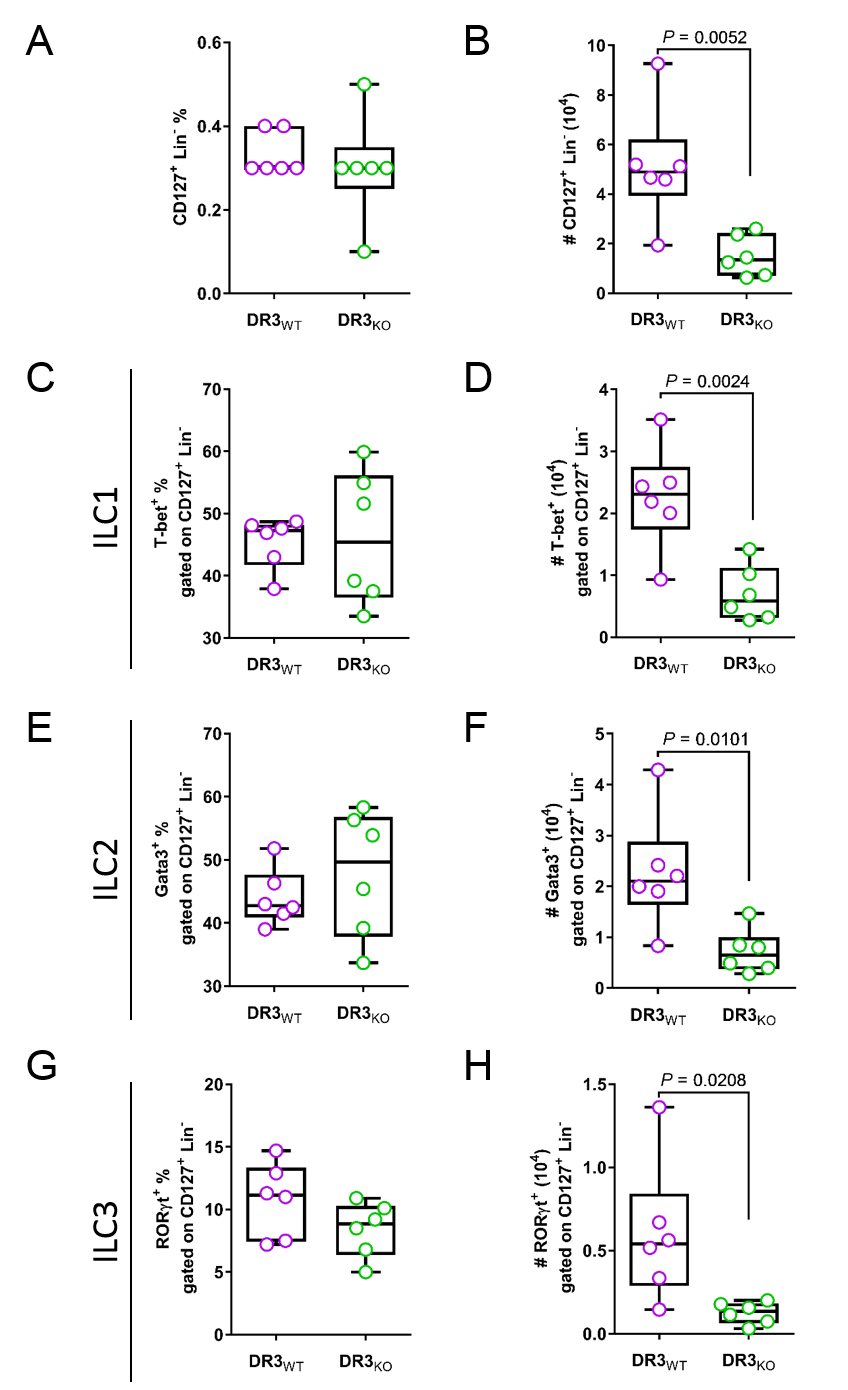

Supplement: Figure S6 — DR3 deficiency is associated with constitutive reduced innate lymphoid cell (ILC) number. Flow-cytometric analysis of mesenteric lymph node cell DR3WT and DR3KO mice (10-week-old, n = 6) after staining with specific Abs for detection of ILC populations, including T-bet+ ILC1, gata-3+ ILC2, and ROR-γt+ ILC3. (A,C,E,G) Cell frequencies and (B,D,F,H) absolute cell numbers presented as median ± interquartile range and analyzed by two-tailed unpaired t-test. Data are representative of three independent experiments. [file Image_6.TIF]
