# Supplementary material for: Death Receptor 3 Signaling Controls the Balance between Regulatory and Effector Lymphocytes in SAMP1/YitFc Mice with Crohn’s Disease-Like Ileitis
Source: Front Immunol. 2018 Mar 1;9:362. doi: 10.3389/fimmu.2018.00362 (PMC5837992; doi:10.3389/fimmu.2018.00362)
Supplement: Table S1 — Characterization of CD25+/–FoxP3+ cell subsets through selected markers in MLNs of unmanipulated AKR, SAMP and DR3KO mice. The results are expressed as the mean percentage ± one SD, and analyzed by 1-way ANOVA with Bonferroni post-hoc test. The number of samples is indicated in parentheses. [file Table_1.DOCX]

| T cell subsets | Frequency of positive cells (percentage) | | | | |
| --- | --- | --- | --- | --- | --- |
|  | AKR  (n=9) | SAMP  (n=9) | DR3_KO_  (n=7) | p value (SAMP vs AKR) | p value (SAMP vs DR3_KO_) |
| **CD3^+^CD4^+^/**MLN/Live | 50.06±11.82 | 35.2±8.366 | 47.79±3.277 | 0.0030 | 0.0138 |
| **FoxP3^+^CD25^+^**/CD3^+^CD4^+^ | 9.878±1.581 | 10.68±2.76 | 9.917±1.854 | 0.8716 | 0.9750 |
| **IL-10^+^/**FoxP3^+^CD25^+^/CD3^+^CD4^+^ | 3.646±1.625 | 6.464±3.216 | 10.31±9.742 | 0.6572 | 0.3720 |
| **CD73^+^**/FoxP3^+^CD25^+^ | 66.72±11.20 | 81.38±6.407 | 77.51±12.44 | 0.0086 | 0.8562 |
| **CTLA4^+^**/FoxP3^+^CD25^+^ | 30.26±14.05 | 52.66±20.03 | 47.69±14.98 | 0.0155 | >0.9999 |
| **Icos^+^**/FoxP3^+^CD25^+^ | 13.09±9.423 | 22.64±7.908 | 21.78±5.87 | 0.0302 | >0.9999 |
| **Nrp-1^+^**/FoxP3^+^CD25^+^ | 32.76±5.528 | 52.24±13.84 | 49.91±14.96 | 0.0037 | >0.9999 |
| **Helios^+^**/FoxP3^+^CD25^+^ | 57.42±7.187 | 50.94±3.814 | 51.46±10.04 | 0.1247 | >0.9999 |
| **PD-1^+^**/FoxP3^+^CD25^+^ | 98.54±1.811 | 99.43±0.6533 | 99.39±0.6198 | 0.2242 | >0.9999 |
| **GITR^+^**/FoxP3^+^CD25^+^ | 77.38±14.22 | 89.58±8.423 | 87.1±11.62 | 0.0610 | >0.9999 |
| **CD103^+^**/FoxP3^+^CD25^+^ | 11.92±8.762 | 23.58±12.66 | 20.33±13.62 | 0.0842 | >0.9999 |
| **DR3^+^**/FoxP3^+^CD25^+^ | 39.45±23.77 | 46.77±23.25 | 1.905±1.268 | 0.8736 | 0.0002 |
| **ROR-γt^+^**/FoxP3^+^CD25^+^ | 65.1±35.850 | 59.43±21.81 | 70.55±20.83 | >0.9999 | 0.8208 |
| **DR3^+^**/ROR-γt**^+^**/FoxP3^+^CD25^+^ | 47.01±15.77 | 52.86±12.58 | 3.629±5.726 | 0.6488 | <0.0001 |
| **Gata3^+^**/FoxP3^+^CD25^+^ | 92.69±14.25 | 96.81±4.411 | 96.6±5.603 | 0.7165 | >0.9999 |
| **DR3^+^**/Gata3^+^/FoxP3^+^CD25^+^ | 54.36±14.64 | 63.03±7.806 | 1.33±0.885 | 0.1460 | <0.0001 |
| **RORγt^-^Gata3^-^**/FoxP3^+^CD25^+^ | 2.029±3.007 | 1.958±3.313 | 0.935±1.403 | >0.9999 | 0.9035 |
| **FoxP3^+^CD25^-^**/CD3^+^CD4^+^ | 76.96±12.02 | 68.66±10.34 | 65.73±14.22 | 0.3193 | >0.9999 |
| **IL-10^+^/**FoxP3^+^CD25^-^/CD3^+^CD4^+^ | 0.1878±0.09 | 0.5933±0.42 | 1.156±1.608 | 0.7600 | 0.4541 |
| **CD73^+^**/FoxP3^+^CD25^-^ | 14.82±11.66 | 21.07±12.56 | 18.87±12.12 | 0.5465 | >0.9999 |
| **CTLA4^+^**/FoxP3^+^CD25^-^ | 1.311±1.339 | 4.224±6.377 | 3.141±3.039 | 0.3079 | >0.9999 |
| **Icos^+^**/FoxP3^+^CD25^-^ | 0.4567±1.21 | 0.213±0.2414 | 0.2463±0.1165 | 0.9332 | >0.9999 |
| **Nrp-1^+^**/FoxP3^+^CD25^-^ | 0.5022±0.3641 | 1.64±1.081 | 1.083±0.6386 | 0.0078 | 0.2856 |
| **Helios^+^**/FoxP3^+^CD25^-^ | 2.154±0.8662 | 2.937±1.089 | 2.356±0.8798 | 0.1770 | 0.4281 |
| **PD-1^+^**/FoxP3^+^CD25^-^ | 99.87±0.1871 | 99.87±0.2541 | 99.93±0.08864 | >0.9999 | >0.9999 |
| **GITR^+^**/FoxP3^+^CD25^-^ | 6.594±7.677 | 11.09±6.545 | 11.05±7.164 | 0.3633 | >0.9999 |
| **CD103^+^**/FoxP3^+^CD25^-^ | 1.896±1.393 | 1.273±2.044 | 0.9163±1.444 | 0.8556 | >0.9999 |
| **DR3^+^**/FoxP3^+^CD25^-^ | 22.64±14.85 | 36.57±22.98 | 0.9988±0.8702 | 0.1605 | 0.0003 |
| **DR3^+^**/ILC1 |  | 68.5±15.3 | 10.05±15.94 |  | <0.0001^§^ |
| **DR3^+^**/ILC2 |  | 95.47±3.46 | 15.13±27.55 |  | <0.0001^§^ |
| **DR3^+^**/ILC3 |  | 94.32±5.48 | 12.42±20.08 |  | <0.0001^§^ |

^§^ Unpaired two-tailed t-Test
