# Supplementary material for: Death Receptor 3 Signaling Controls the Balance between Regulatory and Effector Lymphocytes in SAMP1/YitFc Mice with Crohn’s Disease-Like Ileitis
Source: Front Immunol. 2018 Mar 1;9:362. doi: 10.3389/fimmu.2018.00362 (PMC5837992; doi:10.3389/fimmu.2018.00362)
Supplement: Supplementary file 8 [file Table_2.DOCX]

**Supplementary Table 2.** Statistical analysis carried out by paired two-tailed t-Test to compare the frequency of cells expressing the indicated markers, within FoxP3^+^CD25^+^ vs FoxP3^+^CD25^-^ subsets.

|  | **FoxP3^+^CD25^+^ vs FoxP3^+^CD25^-^** | | | | | | | | | |
| --- | --- | --- | --- | --- | --- | --- | --- | --- | --- | --- |
|  | **IL-10^+^** | **CD73^+^** | **CTLA-4^+^** | **Icos^+^** | **Nrp-1^+^** | **Helios^+^** | **PD-1^+^** | **GITR^+^** | **CD103^+^** | **DR3^+^** |
| **AKR** | 0.0002 | <0.0001 | <0.0001 | 0.0049 | <0.0001 | <0.0001 | ns | <0.0001 | 0.0108 | 0.0026 |
| **SAMP** | 0.0005 | <0.0001 | <0.0001 | <0.0001 | <0.0001 | <0.0001 | ns | <0.0001 | 0.0001 | 0.0052 |
| **DR3^-/-^×SAMP** | 0.0113 | <0.0001 | <0.0001 | <0.0001 | <0.0001 | <0.0001 | ns | <0.0001 | 0.0033 |  |
